# Comparative Assessment of the Antibacterial Efficacies and Mechanisms of Different Tea Extracts

**DOI:** 10.3390/foods11040620

**Published:** 2022-02-21

**Authors:** Shuyuan Liu, Qiqi Zhang, Hang Li, Zheyu Qiu, Youben Yu

**Affiliations:** College of Horticulture, Northwest Agriculture & Forestry University, Xianyang 712100, China; shuyuan_liu@nwafu.edu.cn (S.L.); zqqzqq@nwafu.edu.cn (Q.Z.); lihang@nwafu.edu.cn (H.L.); qiuzheyu@nwafu.edu.cn (Z.Q.)

**Keywords:** tea extracts, pathogenic bacteria, antibacterial activity, catechins, cell membrane

## Abstract

Tea is a popular beverage known for its unique taste and vast health benefits. The main components in tea change greatly during different processing methods, which makes teas capable of having different biological activities. We compared the antibacterial activity of four varieties of tea, including green, oolong, black, and Fuzhuan tea. All tea extracts showed antibacterial activity and Gram-positive bacteria (*Enterococcus faecalis* and *Staphylococcus aureus*) were more susceptible to tea extracts than Gram-negative bacteria (*Escherichia coli* and *Salmonella typhimurium*). Green tea extracts inhibited bacterial pathogens much more effectively in all four varieties of tea with the minimum inhibitory concentration (MIC) values at 20 mg/mL, 10 mg/mL, 35 mg/mL, and 16 mg/mL for *E. faecalis*, *S. aureus*, *E. coli,* and *S. typhimurium*, respectively. Catechins should be considered as the main antibiotic components of the four tea extracts. Total catechins were extracted from green tea and evaluated their antibacterial activity. Additional studies showed that the catechins damaged the cell membrane and increased cell membrane permeability, leading to changes in the relative electrical conductivity and the release of certain components into the cytoplasm. Tea extracts, especially green tea extracts, should be considered as safe antibacterial food additives.

## 1. Introduction

Increased foodborne illness caused by foodborne spoilage and pathogenic bacteria has been a major food safety challenge and caused widespread concern [1]. Pathogenic bacteria have been reported to pose serious threats to human health, such as causing food poisoning, toxic shock syndrome, and infections [2]. In recent years, the chemical preservatives used in food have been reported causing respiratory illnesses or other health risks [3]. Therefore, there is a need to develop novel types of effective plant-derived antimicrobial compounds with safety, biodegradability, and fewer side effects [4]. Some plant extracts, including tea leaves (*Camellia sinensis* (L.) O. Kuntze), have been reported to display good inhibitory effects against pathogenic bacteria and may support the development of antimicrobial supplements [5,6].

Tea is one of the most widely consumed beverages worldwide and is considered to exert various pharmacological effects, such as hypoglycemic, anti-obesity, anti-carcinogenic, antioxidant, anti-arteriosclerotic, and antibacterial activities [7,8,9]. More than 300 different commercial teas have been produced and consumed world-wide. Generally, based on the manufacturing process, teas can be classified into several groups: non-fermented tea (green tea), semi-fermented tea (oolong tea), fully fermented tea (black tea), and post-fermented tea (dark tea) [10]. The active components that play key roles in most of the biological activities of tea are known to be catechins [11]. The presence of different forms of catechins and their derivatives in different teas made these compounds capable of having different biological activities [12]. Due to being steamed or panned, catechin oxidation is prevented in green tea, and polyphenols are essentially maintained in their monomeric forms, such as (-)-epicatechin (EC), (-)-epicatechin gallate (ECG), (-)-epigallocatechin (EGC), and (-)-epigallocatechin gallate (EGCG) [13]. Black tea leaves are subjected to crushing, withering, and a full fermentation process allowing enzyme-mediated oxidation where catechin derivatives are condensed to form theaflavins and thearubigins [14]. Oolong tea has a processing sequence similar to that of black tea but with a shorter oxidation duration, and contains both catechins and theaflavins [15]. Dark teas are post-fermented under controlled conditions of high humidity and temperature with fungi, which greatly decrease the contents of catechins and form tea pigments such as theabrownins [16]. Fuzhuan tea is a typical dark tea. Special fungal fermentation process of Fuzhuan tea promoted the special fungal flower aroma and mellow taste [17]. Other factors, such as the geographical location, growing conditions, variety, and preparation of the infusion, also probably influence the composition of the tea [18,19,20].

Tea flush extract and extracts of various tea products have shown a wide range of an-timicrobial activities against *Bacillus cereus*, *Campylobacter jejuni*, *Escherichia coli*, *Clostridi-um perfringens*, *Helicobacter pylori*, *Legionella pneumophila*, *Staphylococcus aureus*, *Salmonella typhimurium*, *Shigella flexneri*, *Pseudomonas aeruginosa*, *Vibrio cholerea*, etc. [4,21]. A comparative assessment of antimicrobial activity reported that green tea has greater antimicrobial activity than black tea [22,23,24]. The catechins of green tea are mainly responsible to inhibit bacterial growth. EGCG is the most abundant green tea catechin and shows strong bactericidal activity against methicillin-resistant *S. aureus* and penicillinase-producing *S. aureus* by inhibiting the biosynthesis of β-lactams [25,26]. The harvesting season also influences the antibacterial activity of tea, and oolong tea leaves prepared in the summer have been shown to exhibit the strongest activity, followed by those prepared in the spring, winter, or fall [27]. Catechin levels might be the main factor affecting the antibacterial activity of tea, which is influenced by the degree of fermentation and harvesting season. Some organic acids have shown highly antimicrobial activity against a diverse range of pathogens [28]. Kombucha, fermented from tea leaves, has antibacterial activity mainly due to the present of organic acids, such as the acetic acid [29]. Tea tree essential oil has also been reported to have highly antibacterial properties [30]. However, the precise antibacterial spectrum of tea extracts is still difficult to assess. Therefore, green tea, oolong tea, black tea, and Fuzhuan tea manufactured from the same batch of fresh leaves were used in this study to explore the efficacy and possible mechanisms of antibacterial activity, which will be helpful for developing new bacteriostatic agents.

## 2. Materials and Methods

### 2.1. Microorganisms and Materials

Bacteria stains including *E. faecalis* ATCC29212, *S. aureus* ATCC25923, *E. coli* ATCC25922, and *S. typhimurium* ATCC29213 were obtained from the College of Food Science and Engineering, Northwest Agriculture and Forestry University and maintained in slants of nutrient agar (NA) at 4 °C. All microorganisms were cultured in nutrient broth (NB) at 37 °C for 24 h. NA and NB were procured from Beijing Land Bridge Technology Company Limited (Beijing, China). Catechin standards, gallic acid, and caffeine were purchased from Shanghai Yuanye Biotechnology (Shanghai, China). High-performance liquid chromatography (HPLC) grade methanol, acetonitrile, and trifluoroacetic acid were purchased from Thermo Fisher Scientific (Waltham, MA, USA). Bradford protein assay kits were purchased from the Nanjing Jiancheng Bioengineering Institute (Nanjing, China). All other reagents and solvents were purchased from the China National Pharmaceutical Group Corporation (Beijing, China) and were of analytical grade. Purified water (18.2 MΩ) was prepared using a Millipore Mill-Q Ultrapure Water System (Billerica, MA, USA).

### 2.2. Preparation of the Tea Extracts

**Figure 1:**
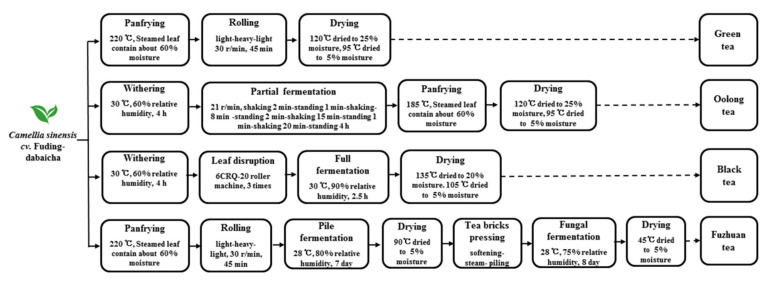
Main manufacture process of various teas.

Fresh tea leaves (*Camellia sinensis cv.* Fuding-dabaicha) were harvested from the tea station at Northwest Agriculture and Forestry University, Yangling, China. The same fresh green shoots were washed thoroughly with water and manufactured into green tea, oolong tea, black tea, and Fuzhuan tea by different manufacturing processes (Figure 1).

Tea extracts were prepared according to the described methods [31]. Briefly, four different varieties tea cut into thin slices and extracted twice with purified boiling water (tea/water *w*/*v* = 1:20) for 7 min. The solutions were collected and concentrated by a RE-52AA vacuum rotary evaporator (Shanghai Yarong Biochemistry Instrument Factory, Shanghai, China), and then freeze-dried (Coolsafe 110-4, Labogene ScanVac, Lynge, Denmark).

### 2.3. Extraction of Catechins

Total catechins (TCs) were isolated from green tea as described previously [32]. The tea leaves were cut into thin slices and extracted twice with 85% ethanol at 35 °C for 30 min. After filtration, the solutions were collected and concentrated. Equal volume of chloroform was added to remove the caffeine, lipids, and chlorophyll. The aqueous phase was extracted with equal volume of ethyl acetate three times. The combined ethyl acetate fractions were collected and freeze-dried to obtain the TCs.

### 2.4. Chemical Identification of Tea Extracts

The total polyphenol content was determined according to the Folin-Ciocalteu method [33]. The amino acid content was assessed according to the China National Standard (B/T 8314-2013, GAQSIQ, China, 2013). The amounts of water soluble carbohydrates were measured by the anthrone-sulfuric acid method [34]. Caffeine, gallic acid, and catechins were identified and quantified by HPLC-UV (Agilent 1100VL, Agilent Technologies Inc., Santa Clara, CA, USA) as described in our previous work [35]. Separations were performed at 35 °C on an Agilent TC-C18 column (5 μm, 250 mm × 4.6 mm i.d.). A gradient mobile phase consisting of water (0.1% formic acid, A) and methanol (0.1% formic acid, B) were eluted as follows: 0–2 min, 80–75% A; 2–6 min, 75–70% A; 6–10 min, 70–75% A; 10–13 min, 75–70% A; 13–20 min, 70% A; 20–23 min, 70–75% A; 23–25 min, 75–80% A; 25–30 min, 80% A, at a flow rate of 1.0 mL/min. The injection volume was 5 µL and detection wavelength was 278 nm. The total theaflavins, thearubigins, and theabrownins were measured according to Zhong [36].

### 2.5. Bacterial Susceptibility Test

The antibacterial activities were evaluated by the disc diffusion method in agar plates according to Goni et al. [37]. Each sterile petri plate (90 mm) was prepared with 15 mL of NA. After solidifying, 100 μL of each culture of bacteria (1.0 × 10^7^ cfu/mL) was spread on the surface of agar plates. A sterile filter paper disc (6 mm) immersed in test solution was placed on the plate followed by incubation at 37 °C for 24 h. Tea extracts were used at the concentrations ranging from 20 mg/mL to 80 mg/mL and TCs were used at concentrations ranging from 1 mg/mL to 10 mg/mL. After incubation, the diameter of the inhibition zone (DIZ) was observed and measured in millimetres (mm), and the experiments were performed in triplicate. Sterile water was used as a control. The experiments were performed in triplicate with triplicate samples.

### 2.6. Determination of the Minimum Inhibitory Concentration (MIC)

The lowest concentration of tea extract to inhibit bacterial growth was determined by the broth dilution method according to a previous method [38]. In brief, 100 μL of each bacterial culture (1.0 × 10^7^ cfu/mL) was inoculated into a test tube containing 5 mL of NB followed by the addition of serially diluted aqueous test solutions at different concentrations. All tubes were incubated at 37 °C for 24 h. The percent inhibition was calculated by measuring the absorbance at 600 nm with a UV-Visible Spectrophotometer (Shanghai, China). The MIC values were determined as lowest concentration at which no growth or turbidity was seen. Negative control contained non-inoculated medium and positive control contained inoculated without extract samples.

### 2.7. Membrane Permeability Assay

The membrane permeability of the bacterial cells was evaluated by measuring the change in electrical conductivity according to the method of Kong et al. [39]. The bacterial cells in their late exponential phase were harvested from NB using a centrifuge at 3000× *g* for 10 min. The cells were washed and re-suspended in 5% glucose until the electric conductivities of the cells were stable. TCs (at the MIC concentration) were added into 5% glucose and measured electrical conductivity with a conductivity meter (SevenEasy, Mettler Toledo, Switzerland).

### 2.8. Membrane Integrity Assay

The membrane integrity of the bacterial cells was evaluated by measuring the release of cell constituents, including nucleic acids, solute sugars, and proteins, into the cell suspension [40]. Bacterial cells in their late exponential phase were harvested from NB using a centrifuge at 3000× *g* for 10 min. The cells were washed and re-suspended in 0.1 M phosphate buffer solution (PBS, pH 7.4) followed by the addition of TCs (at the MIC concentration). After treatment, the suspensions were collected using a centrifuge at 6000× *g* for 5 min. The absorption at 260 nm was measured to determine the change in nucleic acid content by using a UV-visible spectrophotometer (Shanghai, China). The suspension was also collected to determine the concentration of solute sugars according to the anthrone-sulfuric acid method [34] and the concentration of proteins with a Bradford protein assay kit. An untreated sample was used as a control.

### 2.9. Statistical Analysis

All analyses were performed in triplicate using triplicate samples, and the results are expressed as the mean ± standard deviation. Significance differences for multiple comparisons were determined using one-way ANOVA with SPSS (SPSS, Chicago, IL, USA) at *p* < 0.05.

## 3. Results and Discussion

### 3.1. Comparison of the Antibacterial Efficacy of Different Tea Extracts

The antibacterial activity of different tea extracts on Gram-positive (*E. faecalis* and *S. aureus*) and Gram-negative (*E. coli* and *S. typhimurium*) bacteria were evaluated. As shown in Table 1, tea extracts showed varying degrees of antibacterial efficacy against the tested microorganisms in dose-dependent manners at concentrations ranging from 0 to 80 mg/mL. However, different tea extracts exhibited different antibacterial efficiencies. Green tea extracts showed the highest antibacterial activity against both Gram-positive and Gram-negative bacteria, with the largest the DIZ value at the same concentration of all four tea extracts, which was consistent with previous reports [22,41]. Almajano et al. [22] observed that green tea has high inhibitory effect on several microorganisms, and the magnitude of this was comparable to that of commercial infusion. Green tea kombucha has more extensive inhibition on pathogenic bacteria than black tea kombucha [41]. The MIC values of the tea extracts against the test bacteria were consistent with the DIZ values (Table 2). Green tea extracts showed the highest antibacterial activity against the test microorganisms with the lowest MIC values, followed by oolong tea extracts, Fuzhuan tea extracts, and black tea extracts.

Comparing the inhibitory activities of the tea extracts against Gram-negative and Gram-positive bacteria, it was found that tea extracts had stronger antimicrobial activity against Gram-positive bacteria (*E. faecalis* and *S. aureus*) than Gram-negative bacteria. *S. aureus* was the most susceptible bacterium, and *E. coli* was the most resistant bacterium to all tea extracts. The destructive response to the tea extracts of the Gram-positive bacteria compared to the Gram-negative bacteria might be due to the structural differences in the cell walls of the bacteria. The cell walls of Gram-positive bacteria is mainly composed of peptidoglycan and teichoic acid, which are permeable to solute sugars, amino acids, and most ions [42]. However, the cell walls of Gram-negative bacteria are complex and composed of peptidoglycan and lipopolysaccharide outer membranes, which are hard to damage by external factors [1,43]. In addition, the outer membrane of Gram-negative bacteria with a strong negative charge can control the flow of certain substances in and out [42].

### 3.2. Catechins Play the Main Role in Antibacterial Efficacy

In order to clarify the inhibitory mechanism on the antibacterial efficacy of the tea extracts, the basic active components were determined (Table 3). Green tea extracts contained the highest polyphenol (27.10%) and catechin (21.30%) contents, followed by oolong tea extracts (21.60% and 11.16%, respectively), Fuzhuan tea extracts (19.60% and 2.22%, respectively), and black tea extracts (17.26% and 1.67%, respectively). The components of catechins in different tea extracts are different (Table S1). During the fermentation process, large amounts of catechins are reduced and oxidized to form tea pigments [12]. The highest contents of theaflavin (1.80%), thearubigins (16.13%), and theabrownins (17.80%) were detected in the black tea extracts, followed by those in oolong tea extracts (0.87%, 3.13%, and 3.19%, respectively). Due to the special pile-fermenting process and fungal-fermenting process, Fuzhuan tea had a high content of thearubigins (6.76%) and theabrownins (18.77%). The contents of gallic acid and caffeine were the highest in black tea extracts (0.78% and 5.23%, respectively) and Fuzhuan tea extracts (0.75% and 5.48%, respectively), followed by oolong tea extracts (0.55% and 4.12%, respectively), and the contents of these compounds were the lowest in green tea extracts (0.13% and 3.40%, respectively). Carbohydrates were consumed by microorganisms as a carbon source in the special pile-fermenting process and fungal-fermenting process, and the carbohydrate content was the lowest in the Fuzhuan tea extracts (8.91%). There was no significant difference in amino acid content among the four tea extracts.

The catechin components are mainly responsible for several biological activities [44,45]. Line expression analysis showed that the catechin content was positively correlated with the antibacterial capacity of the tea extracts (Table 4). TCs were extracted from green tea extracts, and their antimicrobial activity was determined. As shown in Table 4, the TCs showed dose-dependent antibacterial efficacy against the tested microorganisms at concentrations ranging from 0 to 10 mg/mL. Regarding the different components of bacterial cell wall components, Gram-positive bacteria were more susceptible to TCs than Gram-negative bacteria, with larger DIZ values and lower MIC values (Table 4). Catechin groups are oxidized and polymerized to generate theaflavins, thearubigins, and theabrownins during the fermentation process, and no inhibition zone was observed in the discs containing those tea pigments (data not shown). Therefore, catechins should play the main role in the antibacterial efficacy of all four tea extracts.

**Figure 2:**
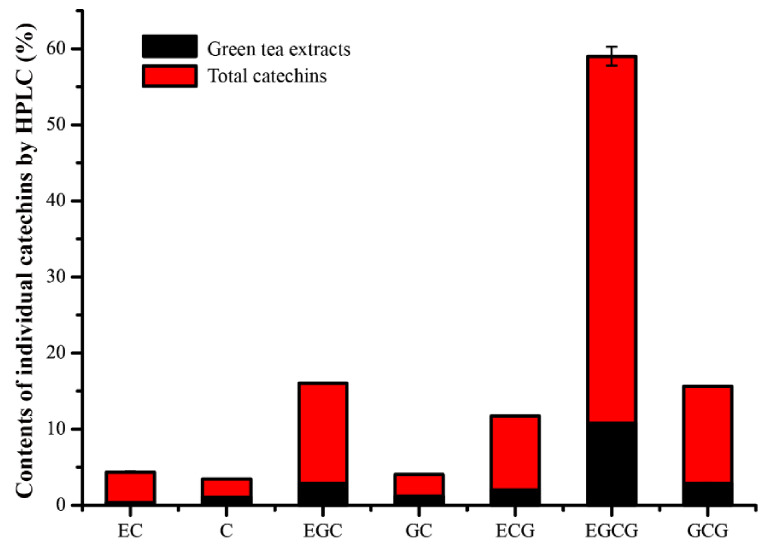
Individual catechin contents determined by HPLC.

HPLC detection and quantitative analysis, as shown in Figure 2, determined the individual TC components. These components were EC (4.03%), (+)-catechin (C, 2.36%), EGC (13.16%), (-)-gallocatechin (GC, 2.87%), EGC (9.73%), EGCG (48.20%), and (+)-gallocatechin gallate (GCG, 12.79%). Comparing the green tea extracts, the TCs had an analogous composition and proportion. EGCG was the main catechin component in both the TCs extracted and the green tea extracts and has been reported to have superior antibacterial effects on *B. anthracis*, *E. coli*, and *S. aureus* [5,27]. Parvez et al. [46] observed that the MIC values of EGCG were 1250 μg/mL against *E. coli* and 625 μg/mL against *S. aureus*. Ignasimuthu et al. [43] measured the MIC values of EGCG for *Bacillus subtilis* (130 μg/mL), *S. aureus* (200 μg/mL), *E. coli* (580 μg/mL), and *Yersinia enterocolitica* (620 μg/mL). Generally, Gram-positive bacteria were more susceptible to EGCG than Gram-negative bacteria.

### 3.3. Antibacterial Mechanism of Catechins

The antimicrobial mechanism of polyphenols is believed to be associated with precipitation of bacterial cell membrane proteins by the reaction of polyphenols [47,48]. Many phenolic acids and flavonoids have been found to cause cell membrane perforation and/or membrane fluidity reduction, leading to cytoplasmic membrane damage [49,50]. To further clarify the antimicrobial mechanism of catechins, the membrane permeability and integrity of TC-treated *E. coli* and *S. aureus* were determined.

**Figure 3:**
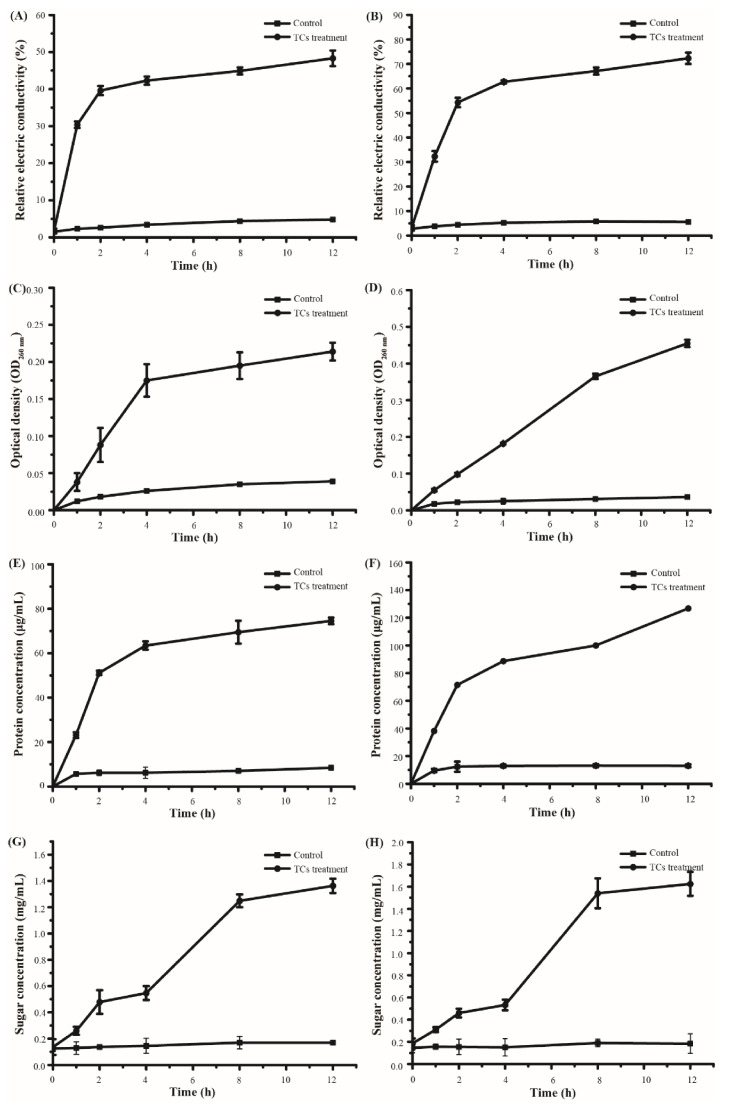
Effects of TCs on the membrane permeability and integrity of *E. coli* and *S. aureus*. (**A**) Changes in the relative electrical conductivity of *E. coli*, (**B**) Changes in the relative electrical conductivity of *S. aureus*, (**C**) Release of nucleic acids from *E. coli*, (**D**) Release of nucleic acids from *S. aureus*, (**E**) Release of proteins from *E. coli*, (**F**) Release of proteins from *S. aureus*, (**G**) Release of solute sugars from *E. coli*, and (**H**) Release of solute sugars from *S. aureus*.

The cell membrane, as a protective barrier for bacteria, will be destroyed by strong antimicrobial agents, leading to internal electrolyte leakage. Changes in relative electrical conductivity were examined as a reflection of the permeability of bacterial cell membranes. As shown in Figure 3A,B, the electrical conductivity for both *E. coli* and *S. aureus* similarly increased after treatment with TCs at their MICs (2.5 mg/mL and 1.5 mg/mL, respectively). The growth trends rapidly increased in response to TCs during the first 2 h, and then the increases slowed down. However, overall, *S. aureus* showed higher electrical conductivity after TC treatment than *E. coli*, indicating that *S. aureus* was more susceptible to catechins increasing the permeability of their cell membrane, causing more cellular leakage.

Membrane integrity was determined by measuring the release of nucleic acids, proteins, and solute sugars after treatment with TCs at their MICs (2.5 mg/mL and 1.5 mg/mL for *E. coli* and *S. aureus*, respectively). The release of intracellular nucleic acids, as an index of cell lysis, can be revealed by changes in optical density at 260 nm [51]. The optical density values at 260 nm increased to 0.214 and 0.455 after *E. coli* and *S. aureus* treatment with TCs, respectively (Figure 3C,D). Organic macromolecule proteins are the basic organics of cells and were shown to be released into the supernatants of the bacteria treated with TCs. More proteins were released into the supernatants of *S. aureus* after TC treatment than in those of treated *E. coli* (Figure 3E,F). Sugar is the primary carbon source and energy storage material for bacteria. The release of intracellular sugar was consistent with the trend of nucleic acids and proteins, which indicated that TCs could lead to sugar leakage through the cell membrane (Figure 3G,H).

Catechins are rich in phenolic hydroxyl groups and polycyclic structures and have a high affinity for biomacromolecules such as lipids, proteins, hydrocarbons, and nucleic acids [48]. This high affinity enables catechins to react with the bacterial cell membrane, which makes the cell structure unstable, alters cell membrane fluidity, and destroys its integrity. These phenomena have been demonstrated by Ignasimuthu et al. [43]. EGCG octaacetate has been found was two-fold higher antibacterial activity against *E. coli* than EGCG, due to its higher lipophilic properties [43]. In our study, the changes in relative electrical conductivity and the release of nucleic acids, proteins, and solute sugars from TC-treated *E. coli* and *S. aureus* were measured. The membrane permeability and integrity changed after treatment with TCs, which indicated that the antimicrobial mechanism of catechins was associated with membrane damage and caused subsequent leakage of the intracellular constituents.

Generally, the membrane permeability and integrity of *S. aureus* were more susceptible to catechins than *E. coli*, which might be partly responsible for the stronger antibacterial activity of catechins against *S. aureus* than *E. coli*. Catechins have been reported to fail to cross the lipopolysaccharide layers of Gram-negative bacteria but exhibit antibacterial activity by binding to the peptidoglycan layer of Gram-positive bacteria [42,43]. The structural differences between Gram-positive and Gram-negative bacteria should be the main reason for the difference in the antibacterial activity of catechins.

## 4. Conclusions

This study compared the antibacterial activity of four kinds of tea extracts (green, oolong, black, and Fuzhuan tea) which were manufactured from the same fresh green shoots with different manufacturing processes. Green tea contained the highest the catechins and showed the best antibacterial activity against Gram-positive bacteria (*E. faecalis* and *S. aureus*) and Gram-negative bacteria (*E. coli* and *S. typhimurium*). Catechins should be considered as the main antibacterial active ingredient in tea extract, and their antibacterial activity were confirmed by test DIZ and MIC values against the test bacteria. The membrane permeability and integrity of *E. coli* and *S. aureus* changed after treatment with catechins. The results suggest that the antibacterial activity of tea extracts is due to the interaction of catechins with the bacterial cell membrane.

## Figures and Tables

**Table 1:** DIZ of tea extracts against tested bacteria.

Test Solution		DIZ (mm)			
		*E. coli*	*S. typhimurium*	*E. faecalis*	*S. aureus*
Green tea	20 mg/mL	6.60 ± 0.19 ^c^	8.80 ± 0.15 ^c^	8.50 ± 0.16 ^a^	21.16 ± 0.08 ^c^
	40 mg/mL	7.20 ± 0.09 ^b^	14.25 ± 0.19 ^b^	19.80 ± 0.22 ^b^	24.50 ± 0.16 ^b^
	80 mg/mL	12.40 ± 0.17 ^a^	22.00 ± 0.13 ^a^	25.90 ± 0.13 ^a^	31.33 ± 0.12 ^a^
Oolong tea	20 mg/mL	6.10 ± 0.04 ^c^	7.20 ± 0.10 ^c^	7.50 ± 0.06 ^c^	18.15 ± 0.12 ^c^
	40 mg/mL	6.70 ± 0.11 ^b^	12.65 ± 0.07 ^b^	16.90 ± 0.11 ^b^	22.60 ± 0.13 ^b^
	80 mg/mL	11.40 ± 0.17 ^a^	20.00 ± 0.11 ^a^	21.90 ± 0.09 ^a^	27.33 ± 0.17 ^a^
Black tea	20 mg/mL	6.10 ± 0.08 ^b^	6.10 ± 0.07 ^c^	6.40 ± 0.05 ^c^	12.02 ± 0.09 ^c^
	40 mg/mL	6.30 ± 0.13 ^b^	9.90 ± 0.03 ^b^	14.80 ± 0.08 ^b^	18.33 ± 0.16 ^b^
	80 mg/mL	10.10 ± 0.04 ^a^	15.30 ± 0.11 ^a^	20.00 ± 0.13 ^a^	25.50 ± 0.13 ^a^
Fuzhuan tea	20 mg/mL	6.10 ± 0.18 ^b^	6.30 ± 0.13 ^c^	7.10 ± 0.13 ^c^	14.33 ± 0.11 ^c^
	40 mg/mL	6.40 ± 0.21 ^b^	10.75 ± 0.12 ^b^	16.50 ± 0.16 ^b^	20.83 ± 0.08 ^b^
	80 mg/mL	11.93 ± 0.10 ^a^	17.10 ± 0.14 ^a^	20.80 ± 0.08 ^a^	25.17 ± 0.17 ^a^

Different small letters in the same column indicate a significant difference at *p* < 0.05 level.

**Table 2:** MIC of tea extracts against tested bacteria.

Test Solution	MIC (mg/mL)			
	*E. coli*	*S. typhimurium*	*E. faecalis*	*S. aureus*
Green tea	35	16	20	10
Oolong tea	45	20	32	12
Black tea	65	36	40	16
Fuzhuan tea	45	38	32	18

**Table 3:** The main constituents of different tea extracts (%).

Tea Extracts	Polyphenol	Catechin	Gallic acid	Caffeine	Carbohydrate	Amino Acid	Theaflavin	Thearubigin	Theabrownin
Green tea	27.10 ± 0. 05 ^a^	21.30 ± 0. 05 ^a^	0.13 ± 0.01 ^c^	3.40 ± 0.02 ^c^	10.41 ± 0.10 ^b^	4.88 ± 0.16 ^a^	0.09 ± 0.01 ^d^	2.71 ± 0.04 ^d^	1.70 ± 0.02 ^d^
Oolong tea	21.60 ± 0.04 ^b^	11.16 ± 0.03 ^b^	0.55 ± 0.02 ^b^	4.12 ± 0.01 ^b^	10.24 ± 0.07 ^b^	4.64 ± 0.24 ^a^	0.87 ± 0.01 ^b^	3.13 ± 0.01 ^c^	3.19 ± 0.06 ^c^
Black tea	17.26 ± 0.03 ^c^	1.67 ± 0.01 ^d^	0.78 ± 0.04 ^a^	5.23 ± 0.02 ^a^	13.24 ± 0.01 ^a^	4.73 ± 0.11 ^a^	1.80 ± 0.01 ^a^	16.13 ± 0.01 ^a^	17.80 ± 0.06 ^b^
Fuzhuan tea	19.60 ± 0.08 ^d^	2.22 ± 0.05 ^c^	0.75 ± 0.004 ^a^	5.48 ± 0.05 ^a^	8.91 ± 0.27 ^c^	4.43 ± 0.37 ^a^	0.24 ± 0.02 ^c^	6.76 ± 0.06 ^b^	18.77 ± 0.15 ^a^

Different small letters in the same column indicate a significant difference at *p* < 0.05 level.

**Table 4:** DIZ and MIC of TCs extracted from green tea against tested bacteria.

Microorganisms	DIZ (mm)			MIC (mg/mL)	Correlation
	1.0 mg/mL	5.0 mg/mL	10.0 mg/mL		
*E. coli*	6.30 ± 0.04 ^b^	11.50 ± 0.16 ^d^	12.20 ± 0.29 ^d^	2.5	0.9866
*S. typhimurium*	6.30 ± 0.05 ^b^	14.30 ± 0.19 ^c^	24.60 ± 0.18 ^c^	2.0	0.9946
*E. faecalis*	7.30 ± 0.05 ^b^	17.90 ± 0.08 ^b^	27.20 ± 0.09 ^b^	2.0	0.9791
*S. aureus*	9.90 ± 0.22 ^a^	22.20 ± 0.10 ^a^	32.20 ± 0.32 ^a^	1.5	0.9997

Different small letters in the same column indicate a significant difference at *p* < 0.05 level.

## Data Availability

The data presented in this study are available within the article.

## Supplementary Materials

The following are available online at https://www.mdpi.com/article/10.3390/foods11040620/s1, Table S1: The monomer components of catechin in tea extracts and GTPs (%).
