# Supplementary material for: Comparative Assessment of the Antibacterial Efficacies and Mechanisms of Different Tea Extracts
Source: Foods. 2022 Feb 21;11(4):620. doi: 10.3390/foods11040620 (PMC8870964; doi:10.3390/foods11040620)
Supplement: Supplementary file 1 [file foods-11-00620-s001.zip › foods-1590461-supplementary.pdf]

Table S1. The monomer components of catechin in tea extracts and GTPs (%).

Tea extracts	GC	EGC	C	EC	GCG	EGCG	ECG
Green tea	1.21±0.05[a]	2.88±0.06[a]	1.10±0.03[a]	0.35±0.04[a]	2.88±0.07[a]	10.80±0.09[a]	2.02±0.05[a]
Oolong tea	1.35±0.04[a]	1.61±0.03[b]	0.55±0.01[b]	ND	2.13±0.07[b]	4.72±0.24[b]	0.78±0.01[b]
Black tea	0.88±0.03[b]	ND	ND	ND	0.28±0.08[c]	0.52±0.04[d]	ND
Fuzhuan tea	0.42±0.02[c]	0.79±0.13[c]	ND	0.14±0.08[b]	ND	0.86±0.06[c]	ND

Note: Data are presented as mean ± SD (n=3). Epigallocatechin-3-gallate (EGCG), epigallocatechin (EGC), epicatechin-3-gallate (ECG), epicatechin (EC), gallocatechin-3-gallate (GCG), gallocatechin (GC), catechin (C). Values with different letters differ from each other significantly ($p < 0.05$). ND means not detected.
